# Hydrochloric acid-treated *Bacillus subtilis* ghosts induce IL-1 beta, IL-6, and TNF-alpha in murine macrophage

**DOI:** 10.1007/s13273-022-00221-5

**Published:** 2022-01-18

**Authors:** Young-Min Kim, Kwang-Su Lee, Won-Mun Kim, Min Kim, Han-Oh Park, Chang Won Choi, Joong-Soo Han, Shin-Young Park, Ki-Sung Lee

**Affiliations:** 1 Department of Biology and Medicinal Science, Pai Chai University, Daejeon, Republic of Korea; 2 Biomedical Research Institute and Department of Biochemistry and Molecular Biology, College of Medicine, Hanyang University, Seoul, Republic of Korea; 3 New Drug R&D Center, Bioneer Corporation, Daejeon, Republic of Korea

**Keywords:** *Bacillus subtilis*, Bacterial ghosts, Hydrochloric acid, Macrophages, Cytokines

## Abstract

**Background:**

Bacterial ghosts (BGs) are empty cell envelopes commonly generated using Gram-negative bacteria; they represent a potential platform for efficient adjuvant and vaccine delivery systems. However, the efficient production of BGs from bacteria in a short period of time is challenging.

**Objective:**

The purpose of this study was to investigate the possibility of producing BGs in the Gram-positive *Bacillus subtilis* using various chemicals, and the potential application of BGs as a novel immunomodulatory agent.

**Results:**

In this study, *Bacillus subtilis* ghosts (BSGs) were generated, for the first time to the best of our knowledge, using the minimum inhibitory concentration (MIC) of hydrochloric acid (HCl; 6.25 mg/mL), sulfuric acid (H_2_SO_4_; 3.125 mg/mL), and nitric acid (HNO_3_; 6.25 mg/mL). Among the BSGs generated using these chemicals, HCl-induced BSGs were completely DNA-free as confirmed by real-time polymerase chain reaction. Scanning electron microscopy showed the formation of transmembrane lysis tunnel structures in HCl-induced BSGs. Murine macrophages exposed to the HCl-induced BSGs at a concentration of 1 × 10^5^ CFU/mL showed a cell viability of 97.8%. Additionally, HCl-induced BSGs upregulated the expression of pro-inflammatory cytokines including interleukin (IL)-1β, tumor necrosis factor alpha, and IL-6. Furthermore, we found differences in the protein expression profiles between intact live bacteria and BSGs using two-dimensional electrophoresis coupled with peptide mass fingerprinting/matrix-assisted laser desorption/ionization-time of flight mass spectrometry analysis.

**Conclusion:**

These data suggest that the HCl-induced BSGs may be potentially safe and effective candidates for inactivated bacterial vaccines and/or immunostimulants.

**Supplementary Information:**

The online version contains supplementary material available at 10.1007/s13273-022-00221-5.

## Introduction

Bacterial ghosts (BGs) are empty bacterial envelopes of Gram-negative bacteria produced via controlled expression of the cloned lysis gene *E* of bacteriophage phi174 (Hajam et al. 2015) that forms transmembrane lysis tunnel structures on bacterial cell surfaces (Eko et al. 2000). Electron microscopic analyses have revealed non-living whole cell envelopes that lack cytoplasmic contents but maintain cellular morphology similar to that of native bacteria, where the entire cell surface structures contain outer membrane proteins, adhesion proteins, lipids, and lipopolysaccharides (LPS) (Huter et al. 1999). BGs represent a potential platform for efficient adjuvant and vaccine delivery systems (Hajam et al. 2017). This approach shows promising results for eliciting immune responses against specific infections in natural hosts as well as in experimental animals (Lubitz et al. 2009). Moreover, the use of whole cells of killed bacteria as a potential vaccine may result in the introduction of antibiotic resistance genes or pathogenic islands into host microbes (Frosch and Meyer 1992). These properties make BGs an attractive tool for vaccine development and antigen delivery systems in both humans and animals (Hajam et al. 2017).

The bacteriophage phi174 lysis gene *E*-induced BGs have been shown to provide efficient protection against specific infections (Tu et al. 2010). However, there are a few disadvantages of this method, such as its application to only Gram-negative bacteria and the potential difficulties in attaining a 100% lysis rate of BGs in a short period (Vinod et al. 2015). Alternatively, novel approaches have been demonstrated for *Escherichia coli* BG preparation using the minimum inhibitory concentration (MIC) and minimum growth concentration of various chemicals (Amara et al. 2013). This protocol requires a short period to generate BGs without any potential risks and is a simple process. Notably, it can be applied to both Gram-positive and Gram-negative bacteria (Vinod et al. 2014). Recently, sodium hydroxide (NaOH)-induced BGs have been produced from a Gram-positive bacterium (*Staphylococcus aureus*) (Vinod et al. 2014) and a Gram-negative bacterium (*Salmonella enteriditis*) (Vinod et al. 2015). Although certain surface structures on BGs may be modified or lost due to NaOH (Park, et al. 2016), the NaOH-induced BGs provide efficient protection against specific infections (Vinod et al. 2017).

*Bacillus subtilis* is a bacterium belonging to the genus *Bacillus* mainly found in soil and is a Gram-positive spore-forming bacterium (Dijl and Hecker 2013). It is also known to be a fermentation microorganism and is used as a microbial agent (Stein 2005). Lactic acid bacteria represent a good source of food additives for immunity enhancement, and they also act as a probiotic agent. However, *B. subtilis* can sometimes cause toxicity, including food poisoning or hepatotoxicity (Poudel et al. 2016). To solve this problem, we produced chemically induced *B. subtilis* ghosts (BSGs), which are dead cells, thereby completely eliminating the toxicity of living cells. In this study, BSGs were successfully produced via chemical-mediated lysis. We investigated the cytotoxic effect of hydrochloric acid (HCl)-induced BSGs on murine macrophage RAW 264.7 cells and their immunomodulatory activities based on the mRNA expression of pro-inflammatory cytokines. Moreover, we analyzed the differences in the protein expression profiles between intact live bacteria and BSGs using two-dimensional electrophoresis (2-DE) and identified the differentially expressed proteins using peptide mass fingerprinting (PMF)/matrix-assisted laser desorption ionization-time of flight (MALDI-TOF) mass spectrometry analysis. This study evaluated the potential efficacy of a chemical-induced BSG vaccine or adjuvant and its ability to induce protective immune responses against pathogens.

## Materials and methods

### Bacterial strain and culture conditions

*Bacillus subtilis* EBM13 (Korean collection for type cultures: 0984BP) was used to produce the BGs. *B. subtilis* was grown in Luria–Bertani broth (LB, pH 7.0) in a shaking incubator (200 rpm) at 36 °C. Bacterial cell growth and lysis were monitored by measuring the absorbance at 600 nm (OD_600_) using a spectrophotometer. The colony-forming units (CFUs) were enumerated as described previously (Park et al. 2016).

### Chemical reagents and determination of MIC

*Bacillus subtilis* cultures were grown to 1 × 10^9^ CFU/mL concentrations in LB broth. Hydrochloric acid (HCl), nitric acid (HNO_3_), and sulfuric acid (H_2_SO_4_) were purchased from Sigma-Aldrich (St. Louis, MO, USA). The lowest concentration of each used chemical was 50 mg/mL. The MIC was determined using the two-fold dilution method (Stickel et al. 2009) with certain modifications. A serially diluted solution of each chemical was added to the bacterial culture, followed by incubation at 36 °C for 18 h. The MICs of the different chemicals were determined in triplicate. To validate the MIC values, the culture that showed no growth was further tested by spreading 100 μL of the culture onto LB agar plates, followed by incubation at 36 °C for 24 h.

### Production of BSGs

The biomass of *B. subtilis* cells cultured for 72 h was pelleted via centrifugation (12,000 × *g* for 15 min at 4 °C), re-suspended twice in phosphate-buffered saline (PBS; 5 mM Na_2_HPO_4_, 150 mM NaCl, 5 mM KH_2_PO_4_, pH 7.0), and used at a final concentration of 1 × 10^9^ CFU/mL. 1 ml of stock solutions (5×) of the different chemicals prepared based on the MIC values was added to 1.8 mL of the bacterial suspension and incubated for 60 min at 36 °C. The rates of cell lysis of the untreated control and bacterial samples treated with the respective chemicals were ascertained at various time points using a standard plating procedure. At the end of the lysis process, the BSGs were harvested via centrifugation (12,000 × *g* for 15 min at 4 °C) and washed thrice with PBS. Finally, cell pellets were re-suspended in PBS and stored at 4 °C until use.

### Isolation and quantification of DNA using real-time polymerase chain reaction (PCR)

To confirm the presence of DNA-free BSGs of *B. subtilis* cells, the bacterial cells were treated with the MICs of different chemicals; non-treated cells were used as the control. Genomic DNA was extracted using a bacterial genomic DNA isolation kit (iNtRON Biotechnology, Gyeonggi-do, Korea). Electrophoresis was performed using a 0.8% agarose gel, and single-step quantitative real-time PCR was performed using the SyBr Green detection system. The 16S rRNA of *B. subtilis* was amplified using specific primers (Table 1). Each 20 μL real-time PCR reaction mix comprised 10 μL of 2 × SyBr Green qPCR Master Mix (Agilent Technologies, Santa Clara, CA, USA), 0.8 μL of each primer (10 pmol/μL), 0.2 μL of Rox dye, 7.2 μL of sterilized distilled water, and 1 μL of template DNA. The thermal cycling conditions were as follows: an initial denaturation at 98 °C for 7 min followed by 39 cycles at 98 °C for 10 s, 56 °C for 30 s, and 72 °C for 30 min in a Stratagene Mx3000P real-time PCR instrument (Agilent Technologies). Negative control TE buffer was included at the same time. All reactions were performed in triplicate. The experiments were analyzed using auto-baseline and thresholds chosen from the real-time PCR amplification. The Ct value and the DeltaRn (dRn) were used for data analysis.

**Table 1:** Primer sequences and fragment sizes of the targeted genes in real-time PCR

Gene	Orientation	Primer sequences (5`-3`)
16 s rRNA (492 bp)	forward	5`-AGAGTTTGATCCTGGCTCAG-3`
	reverse	5`-ATTACCGCGGCTGCTGG-3`
IL-1β (284 bp)	forward	5`-GACCTTCCAGGATGAGGACA -3`
	reverse	5`-AGGCCACAGGTATTTTGTCG -3`
IL-6 (159 bp)	forward	5`-AGTTTGCCTTCTTGGGACTGA -3`
	reverse	5`-TCCACGATTTCCCAGAGAAC-3`
TNF-α (285 bp)	forward	5`-CCGATGGGTTGTACCTTGTC -3`
	reverse	5`-CGGACTCCGCAAAGTCTAAG-3`
GAPDH (165 bp)	forward	5`-GGCATTGCTCTCAATGACAA-3`
	reverse	5`-AGGGCCTCTCTCTTGCTCTC-3`

### Cytotoxicity of macrophages exposed to intact bacteria and BSGs

Murine macrophages (RAW 264.7) were purchased from the Korean Cell Line Bank (ID: 40071; Seoul, South Korea). Cells were cultured in 96-well plates (BD Falcon, Franklin Lakes, USA). The macrophages (1 × 10^5^ cells/mL) were treated with HCl-induced BSGs and live non-treated cells at various doses (1 × 10^5^, 10^6^, 10^7^, 10^8^, and 10^9^ CFU/mL) at 37 °C for 24 h in 5% CO_2_ and 95% air; LPS (5 μg/mL) and PBS were used as the positive and negative controls, respectively. Cell Counting Kit-8 (CCK-8; Sigma-Aldrich) was used to determine cell density. Absorbance (450 nm) was measured using a microplate reader (BioRad Laboratories, Inc., Hercules, CA, USA) and the experiment was performed in triplicate. Cytotoxic activity was calculated using the following formula: cytotoxicity (%) = (1-*A*_450_ of target cells/*A*_450_ of control cells) × 100, where *A*_450_ = absorbance at 450 nm.

### Quantitative analysis of cytokine mRNA expression via quantitative reverse transcription (RT)-qPCR

Raw 264.7 cells were cultured in 24-well plates and treated with the intact *B. subtilis* cells and BSGs separately at a concentration of 1 × 10^5^ CFU/mL. After 24 h of stimulation, total RNA was extracted using the RNAiso Plus kit (Takara Bio Inc., Ohtsu, Japan). cDNA was synthesized via the reverse transcription of 300 ng of purified total RNA using the GoScript^™^ Reverse Transcriptase kit and random primers (Promega Corporation, Madison, WI, USA). qPCR was performed using a SensiFAST^™^ SYBR No-ROX Kit (Bioline, London, UK) on a Stratagene Mx3000P real-time PCR instrument (Agilent Technologies). The primers used for RT-qPCR are listed in Table 1. Thermocycling conditions were 95 °C for 5 min followed by 39 cycles of 95 °C for 30 s and 58 °C for 1 min. Relative quantification was performed using the 2^−ΔΔCt^ method.

### Scanning electron microscopy (SEM)

Morphological features of the HCl-induced BSGs and native *B. subtilis* cells were analyzed via SEM as previously described (Fernandes et al. 1985). Cells were fixed with 2.0% glutaraldehyde in 0.15 M phosphate buffer (pH 7.0) at 4 °C for 2 h. Subsequently, the cells were washed thrice with the same buffer and post-fixed in 1.1% osmium tetroxide at 4 °C for 2 h. The fixed cells were then dehydrated using increasing ethanol concentrations (10–100%) and dried using liquid carbon dioxide. Thereafter, the samples were sputtered with gold–palladium using a high-resolution sputtering system before scanning. Electron micrograph images were examined using a Leo 1455VP scanning electron microscope (Korea Basic Science Institute, Daejeon, Korea).

### 2-DE and image analysis

Urea, thiourea, 3-[(3-cholamidopropyl) dimethyammonio]-1-propanesulfonate (CHAPS), Bradford solution, dithiothreitol (DTT), benzamidine, acrylamide, iodoacetamide, bis-acrylamide acetonitrile, sodium dodecyl sulfate (SDS), and trifluoroacetic acid were obtained from Sigma-Aldrich (electrophoresis grade, ACS reagents, ultrapure). Pharmalyte (pH 3.5–10) was purchased from Amersham Biosciences (Little Chalfont, UK) and IPG DryStrips (pH 4–10 NL) were purchased from Genomine Inc. (Pohang, Kyungbuk, Korea). Modified porcine trypsin (sequencing grade) was purchased from Promega. For protein sample preparation, the cultured cell pellets were washed twice with PBS and sonicated for 8 s using Sonoplus (Bandelin electronic, Berlin, Germany). Samples were homogenized using a motor-driven homogenizer (PowerGen125, Fisher Scientific, Rockford, IL, USA) in a sample lysis solution composed of 2 M thiourea containing 4% (w/v) CHAPS, 8 M urea, 2% (v/v) pharmalyte, 1% (w/v) DTT, and 1 mM benzamidine. Freezing and thawing steps for samples were repeated six times in one day. A bead beater was used for lysis of rigid cells. Proteins were extracted for 1 h with vortexing. After centrifugation at 13,000 × *g* for 1.5 h at 15 °C, the insoluble material was discarded and the soluble fraction was used for two-dimensional gel electrophoresis (2D PAGE).

For the 2D PAGE process, IPG dry strips (pH 4–10 NL IPG, 24 cm) were equilibrated for 14 h with 8 M urea and 2 M thiourea containing 4% CHAPS, 1% pharmalyte, and 1% DTT loaded with 300 µg of sample. Isoelectric focusing (IEF) was performed at 18 °C using a Multiphor II electrophoresis unit and EPS 3501 XL power supply (Amersham Biosciences) by following the manufacturer’s instructions. For IEF, the voltage was linearly raised from 200 to 3000 V during 3.5 h for sample entry followed by a constant 3000 V, with focusing complete after 96 kVh. The strips were incubated for 20 min in equilibration buffer (30 mM Tris–Cl pH 6.8 containing 7 M urea, 1% SDS, and 30% glycerol), first with 2% DTT and subsequently with 2.5% iodoacetamide. Equilibrated strips were inserted into SDS-PAGE gels (20 × 24 cm, 11–16%). SDS-PAGE was performed using a Hoefer DALT 2D system (Amersham Biosciences) following the manufacturer’s instructions. The 2D gels were run at 22 °C for 1500Vh. Subsequently, the 2D gels were silver-stained as described by Oakley et al. (Zhu et al. 2012); however, the fixing and sensitization step using glutaraldehyde was omitted. Quantitative analysis of the images was performed using the PDQuest 7 software (BioRad). All matched spots were checked manually. Spots with a significant differential abundance were selected based on two criteria: *t* test < 0.05 and fold change > 2.0. Selected protein spots showing significant differences were identified via mass spectrometry.

### PMF/MALDI-TOF analysis of differentially expressed proteins (spots)

α-Cyano-4-hydroxycinnamic acid (CHCA), 4-Sulfophenyl isothiocyanate, ammonium bicarbonate, and sodium bicarbonate were purchased from Sigma-Aldrich. PMF was performed as follows: for protein identification via PMF, the protein spots were excised, digested with trypsin (Promega), mixed with CHCA in 60% acetonitrile/0.15% trifluoroacetic acid, and subjected to MALDI-TOF analysis (Microflex LRF 20, Bruker Daltonics, Bremen, Germany) as described by Fernandez et al. (Oakley et al. 1980). The peak list was investigated using Flex Analysis 3.0. The threshold used for peak-picking was as follows: 300 for minimum resolution of monoisotopic mass, 3 for S/N. For protein identification, MASCOT (Matrix Science Ltd., London, UK) search program was used. The following parameters were used for the database search: iodoacetamide (Cys) as a complete modification, trypsin as the cleaving enzyme, oxidation (Met) as a partial modification, monoisotopic masses, and a mass tolerance of ± 0.2 Da. The PMF acceptance criteria used was probability scoring.

### Statistical analysis

In each experiment, all measurements were performed at least in triplicate. All quantitative data are expressed as the mean ± standard error of the mean. Data were analyzed using a two-tailed Student’s *t* test, and values of *p* < 0.05 were considered significant. Graphing was conducted with SigmaPlot 12.5 (Systat Software, Inc., San Jose, CA, USA).

## Results

### Production of chemical-induced BGs

**Fig. 1:**
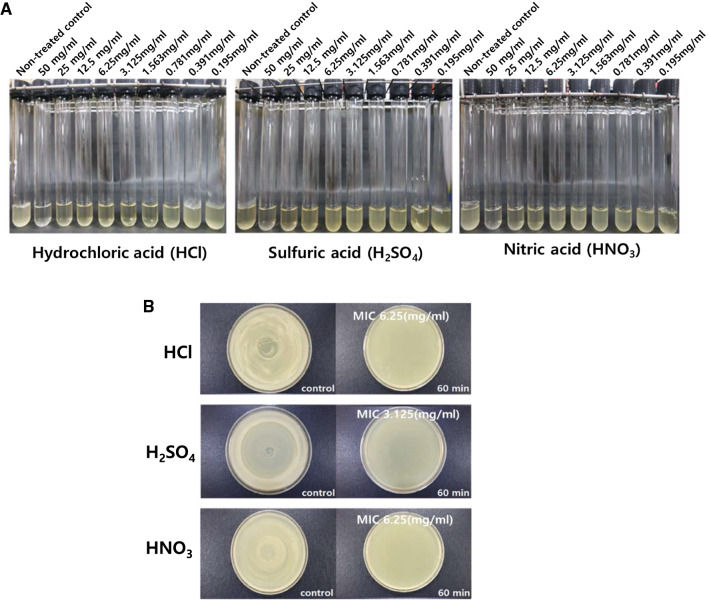
Determination of MIC of different chemicals on *Bacillus subtilis* bacterial cells. (**A**) Tubes 1–9 represent bacterial culture treated with respective chemicals at 0 (non-treated control), 50, 25, 12.5, 6.25, 3.125, 1.563, 0.781, 0.391, and 0.195 mg/ml, respectively. (**B**) *Bacillus subtilis* bacterial cells treated with MIC of the different chemicals showed no viability

To produce the chemical-induced BGs, the MICs of HCl, H_2_SO_4_, and HNO_3_ against *B. subtilis* were first determined using the two-fold broth dilution method (Fig. 1A and Table 2). We found that *B. subtilis* did not form any colonies on LB agar plates on treatment with the chemicals at their corresponding MICs (Fig. 1B), indicating that the MIC of the BGs was determined based on cell death within 60 min of exposure to each chemical. Consequently, this concentration was used to generate non-living BGs.

**Table 2:** Minimum inhibitory concentration (MIC) of chemicals treated into *Bacillus Subtilis* culture medium

Chemical	MIC (mg/ml)	pH (media-MRS)
Hydrochloric acid	6.25 (mg/ml)	1.44
Sulfuric acid	3.125 (mg/ml)	1.46
Nitric acid	6.25 (mg/ml)	1.40

### Determination of DNA-free BSGs

**Fig. 2:**
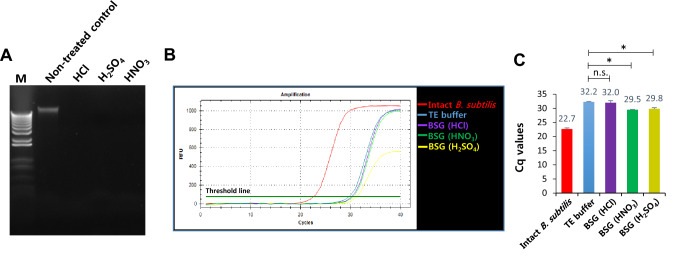
Determination of the most effective chemical for the preparation of DNA-free BSGs. (**A**) Agarose gel (0.8%) electrophoresis of genomic DNA extracted from BSGs treated with the MICs of HCl, H_2_SO_4_, and HNO_3_, for 60 min. M, 1 kb DNA ladder. (**B**, **C**) DNA quantity of the respective BSG was compared with that of intact *B. subtilis* (positive control) and TE buffer (negative control). A standard curve for the absolute quantification of bacterial DNA was generated based on the dilution of genomic DNA of *B. subtilis* in a solution containing 1 ng DNA. All experiments were performed in triplicate (*n* = 3). *p < 0.05 compared with TE buffer (negative control)

A careful consideration of the chemicals used to induce complete DNA-free BGs is necessary to prevent the risk of toxicity. As shown in Fig. 2A, B. *subtilis* cells treated with the MIC of HCl, H_2_SO_4_, or HNO_3_ showed no genomic DNA bands. To confirm this result, we performed qPCR analysis of the BSGs induced by the chemicals (Fig. 2B). Among them, only HCl-induced BSGs showed a complete absence of DNA that was approximately equivalent to the negative control TE buffer (Fig. 2C). This result suggested that DNA-free BSGs were successfully induced using HCl at MIC, and that HCl was the most effective chemical for the production of BSGs.

### Cytotoxicity analysis of murine macrophages exposed to BSGs

**Fig. 3:**
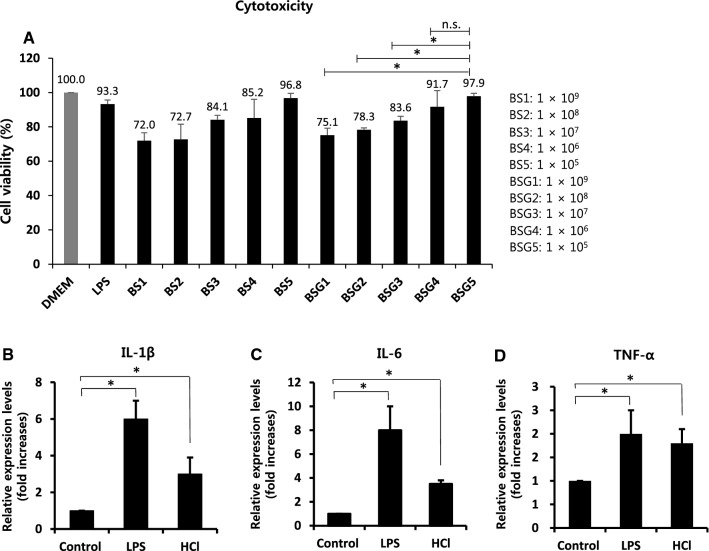
Cell viability and pro-inflammatory cytokine production in BSG-exposed murine macrophage RAW 264.7 cells. (**A**) Macrophages were exposed to DMEM, LPS, intact *Bacillus subtilis* (BS1-5), and BSGs treated with HCl for 60 min (BSG1-5), respectively. At 24 h post-exposure, the macrophages were collected for analysis of cell viability, performed using the Cell counting Kit-8. Bars represent exposure doses of 1 × 10^9^ (BS1 and BSG1), 1 × 10^8^ (BS2 and BSG2), 1 × 10^7^ (BS3 and BSG3), 1 × 10^6^ (BS4 and BSG4), and 1 × 10^5^ (BS5 and BSG5) CFU/mL. Absorbance was measured at 450 nm and all experiments were performed in triplicate. Cytotoxic activity is expressed as the percentage of cell viability using the formula: Cytotoxicity (%) = (1-*A*_450_ of target cells/*A*_450_ of control cells) × 100. The results are based on three independent experiments (*n* = 3). *p < 0.05 compared with BSG5. (**B**–**D**) At 6 h post-exposure of the macrophages to LPS, intact *B. subtilis*, and HCl-induced BSGs (1 × 10^5^ CFU/mL) for 60 min, the macrophages were examined for gene expression of cytokines IL-1β, TNF-α, and IL-6 using RT-qPCR. The results are based on three independent experiments (*n* = 3). *p < 0.05 compared with control. BSG, *Bacillus subtilis* ghost; DMEM, Dulbecco’s modified Eagle’s medium; LPS, lipopolysaccharide; *A*_450_, absorbance at 450 nm; IL-1β, interleukin 1 beta; TNF-α, tumor necrosis factor alpha; RT-qPCR, quantitative reverse transcription-polymerase chain reaction

The cytotoxicity was compared by analyzing the viability of murine macrophage RAW 264.7 cells exposed to Dulbecco’s modified Eagle’s medium (DMEM), LPS, intact *B. subtilis* (BS), and HCl-induced BSGs (Fig. 3A). Since LPS is well known to activate macrophages (Rossol et al. 2011), we used LPS to compare the cytotoxic effects of HCl-induced BSGs on macrophages. The tested concentration of LPS (5 µg/mL) was enough to induce macrophage activation; the macrophages showed a cell viability of 93.3%. The macrophages exposed to HCl-induced BSG5 (1 × 10^5^ CFU/mL) showed a higher viability than those treated with the other BSGs (BSG4: 1 × 10^6^, BSG3: 1 × 10^7^, BSG2: 1 × 10^8^, and BSG1: 1 × 10^9^ CFU/mL); the results were similar to those of macrophages exposed to BS under the same concentration range. This suggested that the MIC of HCl was not associated with a complete reduction in the cytotoxic effect of BS. Macrophages exposed to BSGs at 1 × 10^5^ CFU/mL concentration showed the highest cell viability (97.9%), whereas those exposed to BSGs at 1 × 10^9^ CFU/mL showed the lowest cell viability (75.1%) compared to the results of other concentrations. Since treatment using the BSGs and BS showed the highest cell viability at a concentration of 1 × 10^5^ CFU/mL, we used this concentration in the following experiments.

### Effect of BG treatment on cytokine expression in macrophages

We analyzed the mRNA expression of pro-inflammatory cytokines in macrophages exposed to HCl-induced BSGs and LPS to determine the immunomodulatory activity of BSGs. LPS is responsible for the observed activation of macrophages leading to cytokine production, cytotoxicity, and oxidative stress (Rossol et al. 2011; Soromou, et al. 2012). Therefore, LPS was used as a positive control for cytokine production of macrophages to check whether BSGs produce cytokines in the present study. In the macrophages exposed to the BSGs (1 × 10^5^ CFU/mL), the mRNA expression of IL-1β, IL-6, and TNF-α was significantly increased compared to that in the non-treated control cells (Fig. 3B–D). Collectively, the results suggested that HCl-induced BSGs might have activated the macrophages to secrete pro-inflammatory cytokines and might act as promising inflammatory regulators of mammalian cells.

### Morphology of HCl-induced BSGs based on SEM

**Fig. 4:**
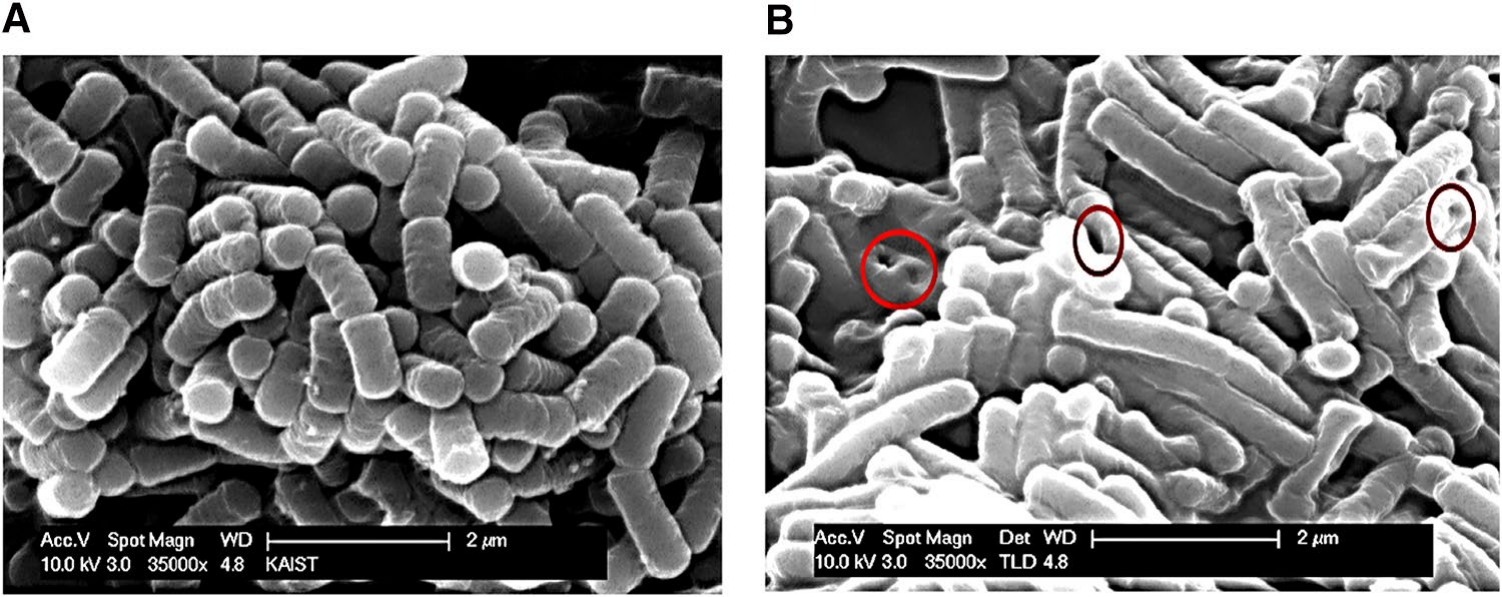
Scanning electron microscopic analysis of BSGs. (**A**) Untreated control shows intact *Bacillus subtilis*. (**B**) Morphology of HCl-induced BSGs. The red circles show the transmembrane lysis tunnels. BSG, *Bacillus subtilis* ghost

We investigated the formation of chemical-induced BSGs and BS using SEM (Fig. 4). When compared with an electron micrograph of untreated BS (Fig. 4A), the electron micrograph of BSGs showed the formation of a transmembrane lysis tunnel structure on the surface of the BSGs (Fig. 4B), indicating that a specific concentration of HCl induced sufficient formation of the transmembrane tunnel structure on the cell surface. This indicated that the cytoplasmic and genetic contents might have been expelled, thereby turning the cell into an empty cell envelope. Except for these pores, BSGs exhibited normal cellular morphology including all cell surface structures that were unaffected by the lysis process.

### Identification of differential protein expression between BS and HCl-induced BSGs

**Fig. 5:**
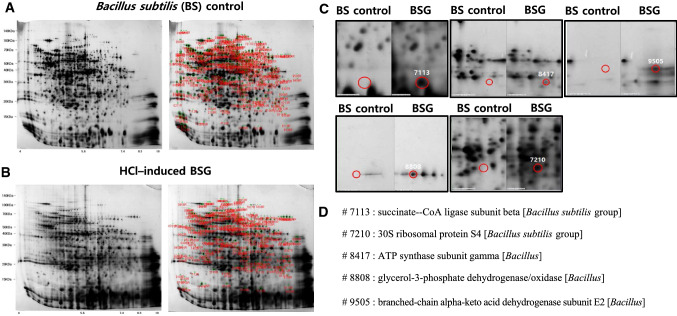
2-DE analysis of the BS control and HCl-induced BSGs. (**A**) Representative 2D-PAGE gels of (**A**) BS control and (**B**) HCl-induced BSGs. Spots indicating significant changes in the expression of the corresponding proteins are labeled by numbers. (**C**) The red circles indicate the five-most increased spots of the HCl-induced BSGs compared to those of the BS control and (**D**) the spots analyzed using MALDI-TOF mass spectrometry. 2-DE, two-dimensional electrophoresis; BS, intact *Bacillus subtilis*; 2D-PAGE, two-dimensional polyacrylamide gel electrophoresis; BSG, *Bacillus subtilis* ghost; MALDI-TOF, matrix-assisted laser desorption/ionization-time of flight

The differences in protein patterns and profiles between the BS control and the HCl-induced BSGs were observed in the 2-DE gel (Fig. 5). In the gel, 840 spots were observed for the BS control (Fig. 5A), whereas 2211 spots were observed for the HCl-induced BSGs (Fig. 5B). After the image analysis, we selected spots (342 spots) of the HCl-induced BSGs (Fig. S1) that showed a significant increase or decrease compared with that of the BS control (≥ twofold, *p* < 0.05). Of those spots, five spots (#7113, #7210, #8417, #8808, and #9205) were of the most increased proteins compared to those of the BS control (Fig. 5C). The five reselected spots were then characterized (Fig. 5D and Table 3). These five proteins were identified as follows: #7113: succinate–CoA ligase subunit beta (*B. subtilis* group), #7210: 30S ribosomal protein S4 (*B. subtilis* group), #8417: ATP synthase subunit gamma (*Bacillus*), #8808: glycerol-3-phosphate dehydrogenase/oxidase (*Bacillus*), and #9505: branched-chain alpha-keto acid dehydrogenase subunit E2 (*Bacillus*). Detailed information on the protein profiles is presented in Fig. S2.

**Table 3:** Several spots changed a lot in both intact cells and BGs

SSP	MW	PI	Spot intensity	
7113	13.47	6.41	1708.78 (BSGs)	1 (BS)
7210	21.49	6.20	713.42 (BSGs)	1 (BS)
8417	34.56	7.54	322.44 (BSGs)	1 (BS)
8808	63.63	7.61	1172.35 (BSGs)	10.88 (BS)
9505	38.93	8.42	1386.14 (BSGs)	1 (BS)

*SSP* standard spot protein, MW molecular weight, *pI* isoelectronic point

## Discussion

The misuse of antibiotics leads to the development of antibiotic-resistant bacteria via a variety of mechanisms, and the spread of antibiotic-resistant bacteria is dangerous to humans (Ventola 2015). To overcome this problem, an alternative approach is to use vaccines. BGs are expected to provide a promising platform for developing new vaccines, combination vaccines or DNA vaccines, including for tumors, and for developing new probiotics (Hajam et al. 2017). The most common method for producing BGs is based on the phage-derived lysis gene *E* (Zhu et al. 2012; Jawale et al. 2012). However, in addition to being restricted to Gram-negative bacteria, it requires a multi-step process that is expensive and time consuming. Therefore, recent studies have demonstrated an alternative method to produce BGs from a Gram-negative or Gram-positive bacterium using the MIC of chemicals, such as NaOH, SDS, and CaCO_3_ (Vinod et al. 2015; Vinod et al. 2014; Amara et al. 2013; Park, et al. 2016; Amro et al. 2014). Similar to the lysis gene *E*-induced BGs, chemical-induced BGs can form the bacterial cell surface transparent membrane structure. It is relatively easy to make vaccines and/or immunity enhancers using this approach, which reduces production cost and labor compared to that required by the genetic engineering method.

The present study successfully generated Gram-positive BSGs. Particularly, BSGs are prepared according to the method used in the present study where cells are treated and cultured with an MIC of HCl capable of inhibiting the growth of bacterial colonies of *B. subtilis*. Additionally, since the BGs are configured such that the shape of the envelope of the cell is intact and the cellular DNA is removed, there is a low risk of adverse effects such as secondary infections due to proliferation, when administered in humans. Of course, *B. subtilis* is a spore-forming bacterium, and its spores can resist heat and chemicals. However, in the course of producing BSG treated with HCl, spore formation was not seen in the photograph of SEM and also there was no colony forming in the plating experiment (for checking whether killed or not) onto the MIC shown in the Fig. 1B. So, there might be no existence of spore materials including sporal DNA. Therefore, BSGs can be effectively used as a vaccine and/or a foreign antigen carrier for the prevention or treatment of infectious diseases caused by Gram-positive *Bacillus* species.

BGs can stimulate the innate immune system without the addition of exogenous adjuvants; BGs are not regarded as genetically modified organisms and have no genetic material (Wang and Lu 2009; Langemann et al. 2010; Muhammad et al. 2012). Therefore, BGs are expected to be superior in terms of safety, and candidate vaccines were found to be highly immunogenic in several studies (Lubitz et al. 2009; Panthel et al. 2003). Moreover, BGs contain well-known innate immune stimulating components such as pro-inflammatory cytokines, and have thus potential to act as efficient adjuvants (Huter et al. 1999; Mayr et al. 2005). In this study, we found that HCl-induced BSGs markedly increased the mRNA levels of IL-1β, IL-6, and TNF-α in murine macrophages, indicating that BSGs are an excellent activator of pro-inflammatory cytokines. In bacterial and viral infections, cytokines play an important role in regulating the host immune system and maintaining the innate immunity (Degre 1996). In the present study, we have confirmed that cytokines are increased by BSGs in macrophages. As inflammation is the basic defense response to various microbial infections (Scheller et al. 1813), we suggest that HCl-induced BSGs have the potential to act as regulators of the innate and adaptive immunity that can defense from bacterial or viral infections. As reported in recent studies, BGs regulate pro-inflammatory factors and chemokines through TLR2 and TLR4 (Quevedo-Diaz et al. 2010; Benko et al. 2008). However, the detailed mechanism of how BSGs increases cytokines in macrophages has not yet been elucidated, further research is needed in future. Furthermore, it is interesting to note that *B. subtilis* elicits an inflammatory response different from that of its diverse BGs produced by different chemical treatments (Hajam et al. 2017; Amara et al. 2013; Lim et al. 2019); therefore, future research in this field should be carried out.

In the present study, by comparing the proteome of the BS control to that of the HCl-induced BSGs using a 2DE-based proteomic approach, we identified five upregulated proteins in the HCl-induced BSGs including succinate-CoA ligase subunit beta, 30S ribosomal protein S4, ATP synthase subunit gamma, glycerol-3-phosphate dehydrogenase/oxidase, and branched-chain alpha-keto acid dehydrogenase subunit E2. These upregulated proteins are mainly related to protein synthesis and cell metabolism. Most importantly, the metabolic activity of energy and protein synthesis are essential to maintaining life. Therefore, the identified proteins upregulated in the HCl-induced BSGs may greatly influence the immune response in the host. Taken together, all of the above results suggest that HCl-induced BSGs may be able to induce an efficient immune response to Gram-positive *Bacillus* infectious diseases, and that it may be useful in future development of novel immunomodulatory agents.

## Supplementary Information

Below is the link to the electronic supplementary material.

Supplementary file1 (DOCX 929 KB)
