# Supplementary material for: Hydrochloric acid-treated Bacillus subtilis ghosts induce IL-1 beta, IL-6, and TNF-alpha in murine macrophage
Source: Mol Cell Toxicol. 2022 Jan 18;18(2):267–76. doi: 10.1007/s13273-022-00221-5 (PMC8764320; doi:10.1007/s13273-022-00221-5)
Supplement: Supplementary file 1 — Supplementary file1 (DOCX 929 KB) [file 13273_2022_221_MOESM1_ESM.docx]

**Supplemental Information**

**Chemical-induced *Bacillus subtilis* ghosts as potential immunostimulants**

Young-Min Kim, Kwang-Su Lee, Won-Mun Kim, Min Kim, Han-Oh Park, Chang Won Choi, Joong-Soo Han, Shin-Young Park and Ki-Sung Lee

**
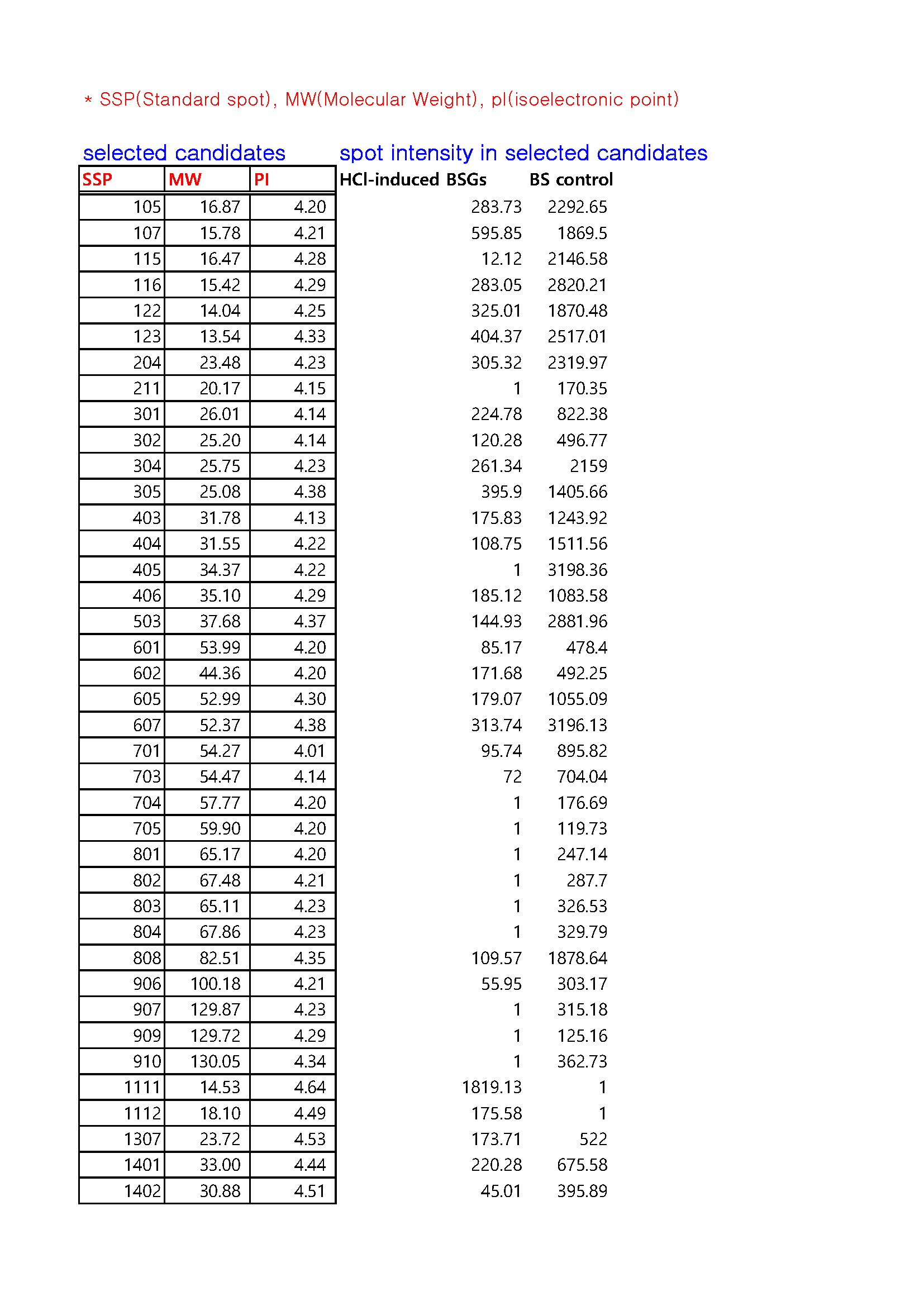
Supplementary Figure 1**

**

**

**

**

**

**

**

**

**

**

**

**

**

Figure S1.** Comparison of Spot intensity between BS control and HCl-induced BSGs in selected candidates from 2-DE images. Quantitative analysis of digitized images was carried out using the PDQuest (version 7.0, BioRad) software according to the protocols provided by the manufacturer. Quantity of each spot was normalized by total valid spot intensity. Protein spots were selected for the significant expression variation deviated over two fold in its expression level compared with BS control or HCl-induced BSGs.

**Supplementary Figure 2**

**
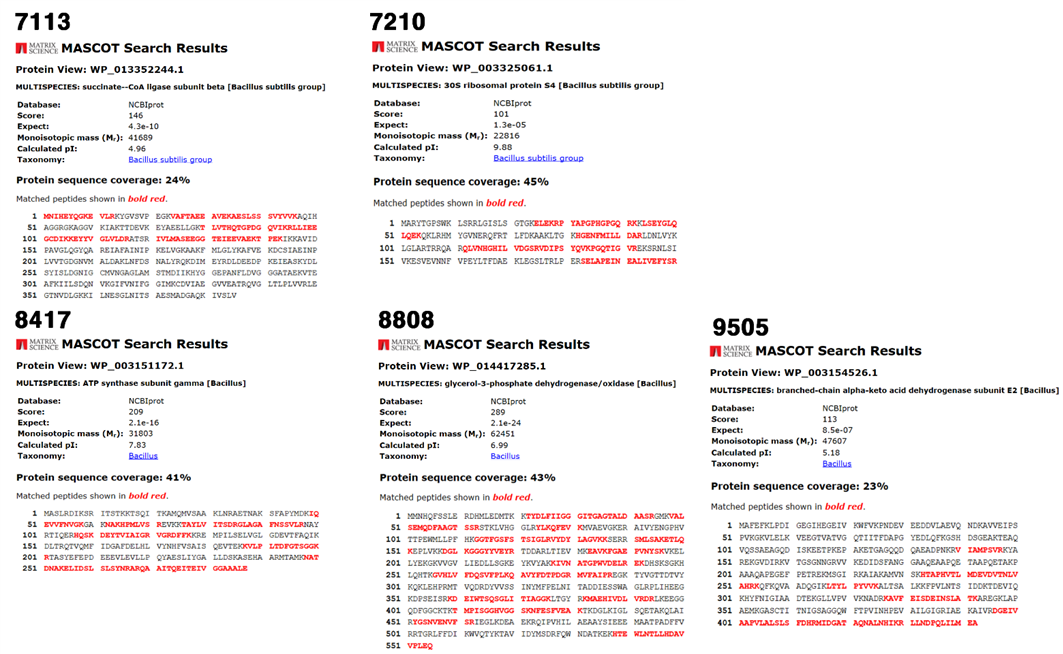
Figure S2.** PMF/MALDI-TOF analysis of specifically changed proteins (spots) in amount. Selected five spots from 2DE PAGE results were identified and characterized.
